# Age-related differences of inter-joint coordination in elderly during squat jumping

**DOI:** 10.1371/journal.pone.0221716

**Published:** 2019-09-09

**Authors:** Sébastien Argaud, Benoit Pairot de Fontenay, Yoann Blache, Karine Monteil

**Affiliations:** 1 Laboratoire Inter-Université de Biologie de la Motricité, Université Lyon, Lyon, France; 2 Centre interdisciplinaire de Recherche en Réadaptation et en Intégration Sociale (CIRRIS), Institut de Réadaptation en Déficiences Physique de Québec (IRDPQ), Université de Laval, Québec, Canada; University of Cassino e Lazio Meridionale, ITALY

## Abstract

**Background:**

Explosive movement requires that the individual exerts force and power with appropriate magnitude and timing. These coordination aspects have received less attention despite being a basic prerequisite for daily mobility and physical autonomy, especially in older people. Therefore, the purpose of this study is to characterize the effect of age on inter-joint coordination during explosive movement.

**Methods:**

Twenty-one elderly and twenty young participants performed three maximal vertical jumps, while kinematics were recorded throughout each squat jump. Inter-joint coordination and coordination variability were calculated for selected sagittal hip-knee, knee-ankle, and hip-ankle joint couplings using the continuous relative phase method.

**Results:**

The young participants produced significantly greater jump height performance (0.36 ± 0.07 m vs. 0.12 ± 0.04 m, *p* < 0.001). The mean absolute continuous relative phase for ankle-knee and knee-hip joint couplings were significantly greater for the elderly in comparison to the young group (p < 0.01 for the both). No significant differences between senior and young participants in the mean absolute continuous relative phase for ankle-hip joint couplings (*p* = 0.25) was observed. However, there was significantly more variability in inter-joint coordination in the elderly marked by greater continuous relative phase variabilities in ankle-knee, ankle-hip and knee-hip joint couplings (*p* < 0.001) than those observed in young adults.

**Conclusion:**

In this study, seniors demonstrated proximodistal inter-joint coordination but with different delays in the pattern of inter-joint coordination during squat jumps compared to young adults. In addition, a higher continuous relative phase variability in the elderly may be needed to improve stability or compensate for strength deficits in jump achievement.

## Introduction

Previous studies have observed a strong association between explosive performance and functional abilities in older adults [1, 2]. For instance, a loss of maximal and explosive force during multi-joint tasks leads to biomechanical impairments that might affect the ability to perform daily activities and cause disability and mortality risk [3–5]. Vertical jumping requires the ability to accelerate a mass as quickly as possible in the shortest time. Though it is not a common daily life activity performed by seniors, the vertical jump is a simple task to investigate maximal power production. In a previous study, lower joint power in the elderly was observed when performing maximal vertical squat jumps [6]. This lesser power resulted from both lower moment and angular velocity produced at each lower limb joint [6–8]. Other studies have indicated that age-related deficits in joint work occurred for all lower limb joints in squat jumping [9, 10]. However, the work distribution among the lower limb joints was not significantly different between old and young adults, contrary to outcomes observed during walking, running, or sprinting activities [11–14].

The dynamic parameters of the lower limbs (i.e. joint power and mechanical work) during explosive tasks are also influenced by inter-joint coordination effectiveness. Indeed, during dynamic tasks in soccer, the preservation of high neuromuscular control is required to limit the decrease in power and interlimb coordination in the elderly [15]. In addition, it was demonstrated that vertical jump height is maximized in the young population when an optimal proximodistal joint extension is performed [16, 17]. By contrast, in the elderly, Haguenauer et al. [18] highlighted a simultaneous lower limb joint extension during the push-off phase of the squat jump. During an explosive leg press task performed as quickly as possible, Wilson et al. [19] observed a similar pattern in the coordination strategies between young and senior populations. In conclusion, the differences in the movement processes and motor patterns due to the effect of age are still unclear in the case of explosive movements.

In addition to the controversial results on inter-joint coordination cited above, Wilson et al. [19] observed a reduced variability in joint coordination strategies in older adults during a leg press task. For these authors, this smaller joint coordination variability may be explained by an inability of seniors to control multiple joints when lower limbs are challenged during fast movement, which would be related to an increase of muscle co-contraction [19]. Greater variability may offer the movement control systems the flexibility to make adjustments in coordination while ensuring successful completion of the intended motor act [20, 21]. Although previous studies have yielded information about the effect of age on inter-joint coordination, to the best of our knowledge, the variability of inter-joint coordination has only been explored during closed-loop tasks, such as a leg press, at relatively low forces, while open-loop tasks, like vertical jumping at body weight, may lead to different results. In this context, the continuous relative phase (CRP) commonly implemented to characterize inter-joint coordination for clinical and sport applications [22–24] could be used to address the lack of literature on the effect of age on inter-joint coordination during vertical jumping.

The purpose of this study was twofold: 1) to characterize inter-joint coordination in the elderly and 2) to determine its variability during the push-off phase of a maximal squat jumping task. It was hypothesized that (1) the elderly display an altered strategy of proximodistal joint extension of the lower limb and (2) display an increased variability in joint coordination strategies.

## Methods

### Participants

Twenty young men (age: 23.1 ± 2.79 years, height: 1.8 ± 0.03 m, mass: 68.8 ± 6.78 kg, body mass index (BMI): 21.8 ± 1.42 kg.m^-2^) and twenty-one older men (age: 74.48 ± 4.6 years, height: 1.70 ± 0.05 m, mass: 79.2 ± 10.2 kg, BMI: 27.2 ± 2.96 kg.m^-2^) volunteered to participate in this study. To differentiate between the two groups, some inclusion criteria were taken into consideration. The seniors had to: a) be over 65 and under 85 years old (World Health Organization [25]); b) actively participate in structured group exercise (Nordic walking, yoga, gymnastics), individual physical activity (gardening, DIY), or sport (cycling, trekking, tennis, boules); and c) engage in at least 150 minutes of moderate-intensity aerobic physical activity throughout the week, or at least 75 minutes of vigorous-intensity aerobic physical activity throughout the week, or an equivalent combination of moderate- and vigorous-intensity activity (determined by a physical activity questionnaire, the PAQUAP [26]). The young participants had to a) be between 18 and 30 years old; b) practice two hours of physical activity per week; and c) not present recent or current musculoskeletal disorders. As proposed by Greig et al. [27], older participants were also selected according to the exclusion criteria to define a standard for ‘‘medically stable” subjects for exercise studies. These exclusion criteria were designed both to ensure safety and to define degrees of freedom from diseases that could impair physical performance. The elderly completed a questionnaire to confirm the absence of a) severe cardiopulmonary and b) neurological impairments; c) balance disorders; or d) recent musculoskeletal disorders. For both groups, the passive range of motion of the ankle, knee, and hip joints was tested to ensure e) the absence of pathological joint mobility [28]. The experimental procedures were performed in accordance with the current laws of the European Union and were approved by the Ethics Committee of the University of Lyon. All participants were carefully informed about the experimental procedures and the possible risks and benefits associated with participation in this study and signed an informed consent.

### Experimental procedure

Before the tests, all participants performed a standardized five-minute warm-up on a cycloergometer (Ergometer X7, Kettler, Germany). Participants also practiced maximal vertical squat jumping with their preferred initial posture to be familiarized with the experimental test. Then, the participants were filmed for four seconds while standing in an upright position for a calibration process. The test consisted of performing three maximal squat jumps with the hands on the hips and without any countermovement [29]. A three-minute rest interval between each trial was implemented to avoid the effects of muscle fatigue. Participants were instructed to jump as high as possible while limiting lateral and horizontal displacement. To enable the detection of the start of the push-off (offline), the participants held the initial posture for one second.

### Instrumentation and data collection

For kinematic analysis, landmarks were located by the same experimenter on the right acromion, greater trochanter, lateral epicondyle of the femur, lateral malleolus, and the fifth metatarsophalangeal joint, according to previous studies analyzing vertical jumping [6, 24]. Squat jumps were filmed in the sagittal plane with a 2D camera operating at 100 Hz (Ueye, IDS UI-2220SE-M-GL; IDS Imaging Development System GmbH, Obersulm, Germany). The optical axis of the camera was perpendicular to the plane of motion, and the lens was located 4 m from the participant. In addition, ground reaction forces were measured with a force platform sampled at 1,000 Hz (AMTI, model OR6-7-2000, Watertown, Massachusetts, USA).

### Data treatment

The dynamic data were smoothed with a zero-lag fourth-order low-pass Butterworth filter with a cutoff frequency of 15 Hz [30] and downsampled to 100 Hz. The presence of any countermovement was visually controlled for each trial. The mean and standard deviation values of the vertical ground reaction force were determined in the first second during which the subject held the initial position. The beginning of the push-off corresponded to the instant after the first second when the vertical ground reaction force increased more than two standard deviation values above the body weight one value [6].

**Fig 1:**
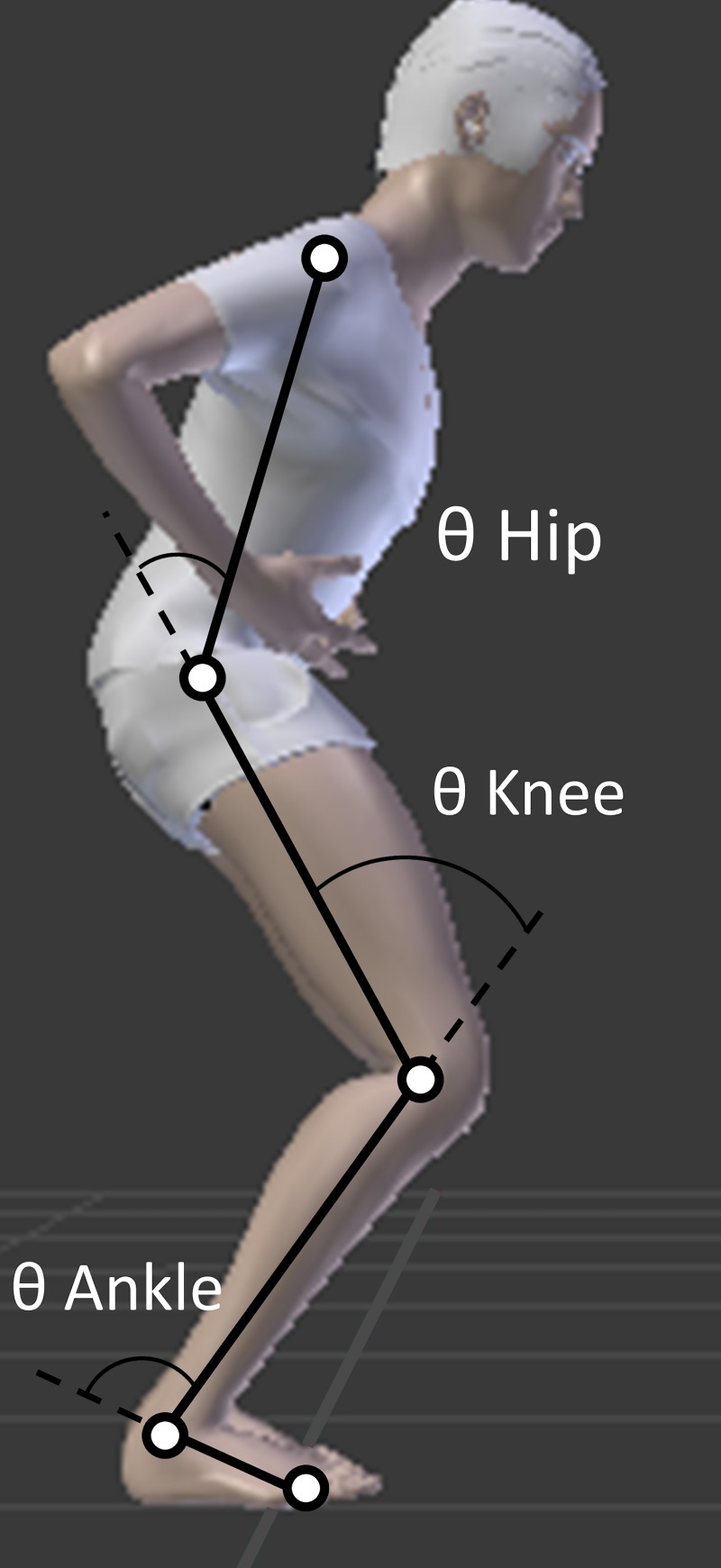
Biomechanical model. Setup of the experimental procedure combined with the kinematic model and the corresponding joint angles.

The reflective markers defined a 2D kinematic model composed of four body segments (feet, lower legs, upper legs, and “head-arm-trunk”) and four degrees of freedom, by gathering the left and right sides (Fig 1). Joint angles were obtained by multibody kinematic optimization to limit the negative effects of soft tissue artifacts [31]. Such an algorithm consists of minimizing the sum of the quadratic difference between the measured and reconstructed marker positions. Finally, the ground reaction force and kinematic signals were synchronized and cut to keep only the push-off phase for analysis.

### Vertical jump height and continuous relative phase

The vertical jump height was defined as the difference between the height of the body mass center (determined from Winter et al. [30]) at the apex of the jump and its height when the participant was standing upright with heels on the ground.

The CRP was calculated between each joint according to Hamill et al. [32] to assess inter-joint coordination during squat jumping. The phase plot was obtained for each joint (*j*) by representing the normalized angular velocity $\left(\dot{\theta }\right)$ with respect to its corresponding normalized angle (*θ*), according to Eq 1 and Eq 2:

$$\theta_{j}^{norm}=\frac{2*\left[\theta_{j}-min\left(\theta_{j}\right)\right]}{max\left(\theta_{j}\right)-min\left(\theta_{j}\right)}$$ Eq 1

$${\dot{\theta }}_{j}^{norm}=\frac{{\dot{\theta }}_{j}}{max\left[max\left({\dot{\theta }}_{j}\right),\max\left(-{\dot{\theta }}_{j}\right)\right]}$$ Eq 2

Then, the phase angle (*φ*) ranging between 0° and 180° was calculated as the four-quadrant arctangent angle formed between the normalized angle velocity and joint angle (Eq 3). Finally, the CRP was calculated between each joint by the difference between the phase angles (Eq 4)

$$\varphi_{j}=\tan^{-1}\left({\dot{\theta }}_{j}^{norm}/\theta_{j}^{norm}\right)$$ Eq 3

$$\begin{array}{l} CRP_{a-k}=\varphi_{ankle}-\varphi_{knee}\\ CRP_{k-h}=\varphi_{knee}-\varphi_{hip}\\ CRP_{a-h}=\varphi_{ankle}-\varphi_{hip} \end{array}]$$ Eq 4

Consequently, the CRP ranged between -180° and 180°. A CRP close to 0° meant that both joints evolved in phase, while a CRP close to 180° or -180° corresponded to an anti-phase[32]. A negative value meant that the proximal joint was leading the distal joint [32]. Finally, two parameters were considered to compare inter-joint coordination between senior and young adults during the push-off phase of a squat jump. Firstly, the mean absolute CRP (MACRP) for each joint coupling was computed [33]. Secondly, for each joint coupling, the standard deviation of the absolute CRP of the three jumps was computed at each time point, and the mean standard deviation was calculated to assess the CRP variability [33].

### Statistics

The difference in vertical jump height between the two groups was tested with independent Student’s t-tests. The linear mixed model was implemented to assess the effect of the group (young vs. elderly) and the joint coupling (knee-hip vs. ankle-knee vs. ankle-hip) on the MACRP and the CRP variability. As within-participants repeated measures were performed for the joint coupling fixed effect, participants were entered as random intercepts. In addition, the BMI of the participants was also entered as a fixed effect to assess whether this parameter may influence the MACRP and CRP variability. The *p*-values were obtained by likelihood ratio tests of the full model against the model without the effect in question. The level of significance was set at *p* < 0.025 (Bonferroni correction: 0.05/2 dependent variables). When a main or interaction effect was revealed by the linear mixed model, post-hoc comparisons were performed using the Tukey’s honestly significant difference procedure. The linearity, homoscedasticity, and normality of the residuals were graphically controlled. For post-hoc comparisons, effect sizes (ES: Cohen’s d) and 95% confidence interval were calculated. The statistics were performed with R software (R 3.2, RCore Team 2014, package *lme4* [34]).

## Results

Young adults jumped, on average, about three times higher than senior adults (0.36 ± 0.07 m vs. 0.12 ± 0.04 m, *p* < 0.001; 95% confidence interval [-0.28, -0.20]; ES: 4.31).

**Fig 2:**
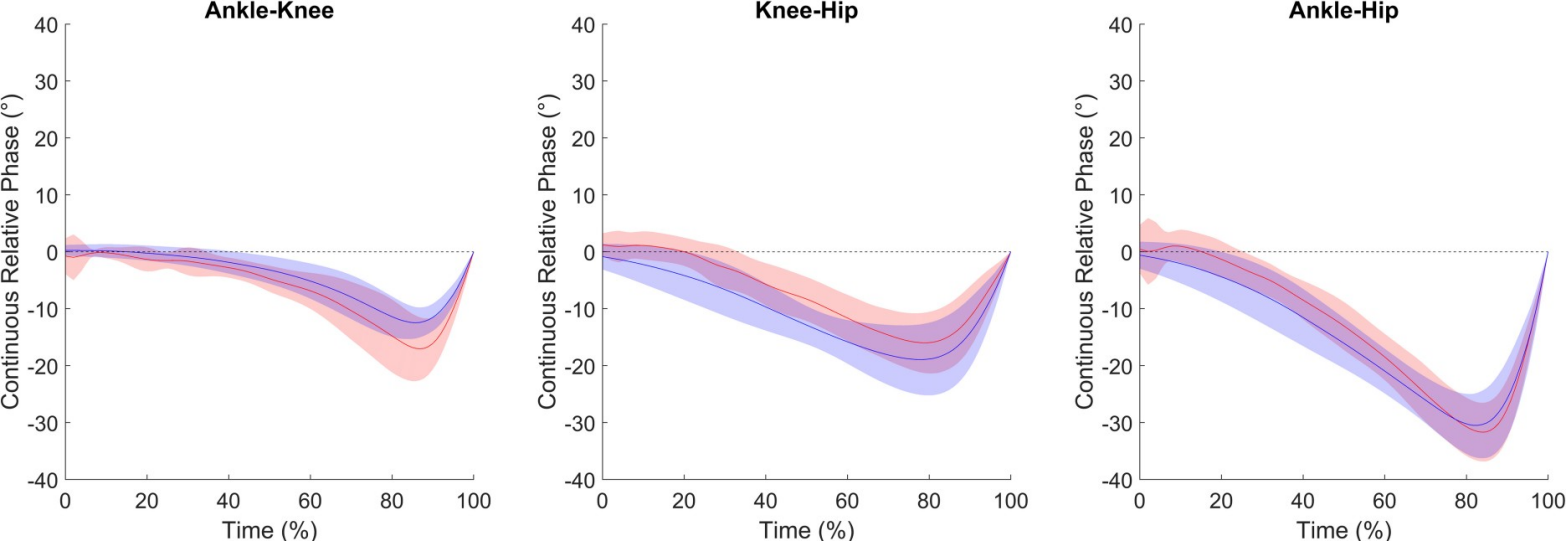
Continuous relative phase. Average curves (blue line for young and red line for elderly groups) and standard deviations (blue and red area) of the continuous relative phase with respect to push-off time. Time equal to 100% corresponds to the takeoff.

The linear mixed model revealed no effect of the BMI on the MARCP, while the interaction effect between the group and joint coupling on the MACRP was observed. Post-hoc tests showed no significant differences between the elderly and young groups for ankle-hip joint couplings (*p* = 0.25; Table 1 and Fig 2). However, the MACRP for ankle-knee and knee-hip joint couplings were significantly higher for the elderly in comparison to the young adult group (*p* < 0.01 for both).

**Table 1:** Mean and Standard deviation of the mean absolute continuous relative phase of the young and elderly groups.

	Elderly	Young	p value	95% CI (E-Y)	ES	Power
ankle-knee	6.3 ± 2.2	4.6 ± 1.6 *	< 0.01	0.43–2.91	0.86	0.78
knee-hip	7.9 ± 2.7	10.5 ± 2.8 *	< 0.01	-4.32 –-0.87	0.95	0.85
ankle-hip	13.9 ± 2.4	14.9 ± 3.0	0.25	-2.72–0.73	0.40	0.24

CI: confidence interval; ES: Effect Size (Cohen’s d)

* means that Elderly are significant different from young adult group

**Fig 3:**
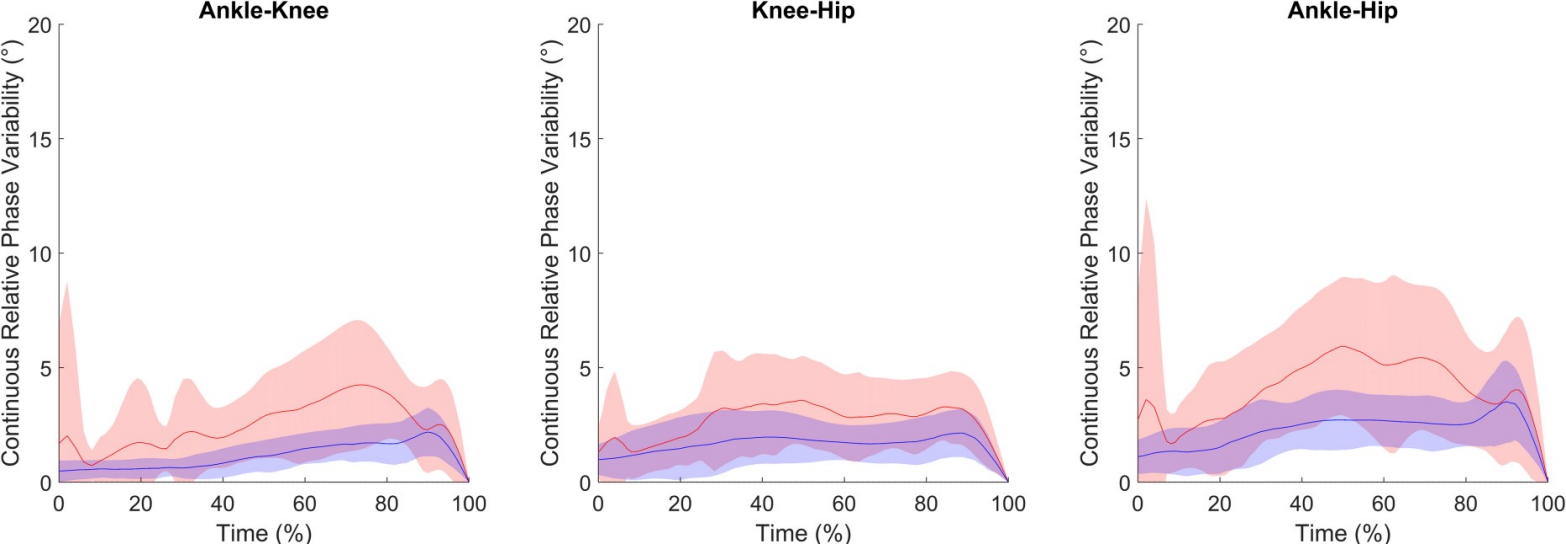
Variability of the continuous relative phase. Average curves (blue line for young and red line for elderly groups) and standard deviations (blue and red area respectively) of the variability of the continuous relative phase with respect to push-off time. Time equal to 100% corresponds to the takeoff.

Finally, the linear mixed model revealed only an interaction effect between the group and joint coupling on the CRP variability. Post-hoc tests indicated that the average CRP variabilities for ankle-knee, ankle-hip, and knee-hip joint couplings in the elderly group were greater (all *p* < 0.01) than those observed in the young group (Table 2 and Fig 3).

**Table 2:** Mean and standard deviation of the continuous relative phase variability of the young (Y) and elderly (E) groups.

	Elderly	Young	p value	95% CI (E-Y)	ES	Power
ankle-knee	2.45 ± 1.2	1.1 ± 0.4 *	< 0.001	0.77–1.89	1.72	0.99
knee-hip	2.6 ± 0.9	1.6 ± 0.5 *	< 0.001	0.54–1.47	1.43	0.99
ankle-hip	4.0 ± 1.8	2.3 ± 0.5 *	< 0.001	0.93–2.62	1.52	0.99

CI: confidence interval; ES: Effect Size (Cohen’s d)

* means that elderly are significant different from young adult group

## Discussion

The purpose of this study was firstly to characterize the inter-joint coordination in the elderly and secondly to determine its variability during the push-off phase of a squat jumping task performed maximally. The main findings were that young adults jumped on average three times higher than seniors. In addition, both the elderly and young participants displayed proximodistal inter-joint coordination but with different delays of joint extension highlighted by different MACRPs. Finally, the elderly performed their squat jumps with higher variability in inter-joint coordination than the young adults.

The first hypothesis of this study was that the proximodistal sequential pattern of joint coordination observed in young adults was altered in the elderly. Contrary to this hypothesis, these findings support that a proximodistal coordination pattern was performed in vertical jumping both for young adults and seniors, as already depicted in young adults [34, 35]. Considering the age-related decline in neuromuscular function, this finding that healthy young and older adults employ proximodistal patterns of inter-joint coordination might be somewhat unexpected. Indeed, elderly, compared with young adults, have deficits in muscle strength [36, 37], muscle power [7, 38], and mobility [12, 13]. They are less able to integrate proprioceptive feedback [39] and to coordinate agonist–antagonist muscle pairs [40], critical in explosive movement tasks. Likewise, Haguenauer et al. [18] exhibited that the initial joint extensions of the hip, knee and ankle occurred simultaneously in seniors during squat jumping. However, the study focused on an older population (mean age 82.6 years vs. 74.48 years) with a great age difference ranged from 79 to 100 years and reduced strength (30% lower jump height). Worsening physical performance was observed as early as the seventh decade [41, 42], which accelerated between 78 and 81 years old [43], and might be associated with reduced function in various daily activities [44]. This study was not designed to test this assumption, but there were significant differences between the 70–80 age range observed in this study and the 80–89+ age population observed in the study by Hagenauer et al. [18]. Nevertheless, these results indicate an alteration in the proximodistal pattern of joint extension when comparing elderly to young adults. The MACRP for knee-hip joint coupling was smaller in the elderly group, meaning that hip joint extension followed the knee joint in comparison to the young group. In contrast, the MARCP for ankle-knee joint coupling was greater in the elderly group, meaning that the knee joint extension preceded the ankle in comparison to the young group. The knee joint’s earlier involvement could be explained by the need for the elderly population to promote a faster increase in center of gravity velocity. In summary, these data suggest an adjustment of motor coordination in healthy seniors, which might be an adaptation to a strength deficit in this population.

The second aim of this study was to characterize the variability in coordination strategies in vertical jumping. The inter-joint coordination variability observed in this study suggested an alteration inside the proximodistal pattern of joint extension [45] and offered information about physical impairment consequences due to the effect of age [46, 47]. A low CRP variability indicated a rigid, stable pattern, while a high CRP variability suggested a more flexible or potentially unstable pattern in inter-joint coordination. In this study, a greater variability of inter-joint coordination was observed in seniors with a greater CRP variability for all joint couplings in comparison to young adults. These results were opposite to those observed in Wilson’s study [19], which investigated inter-joint coordination during a leg press task. These authors observed lower variability in coordination of the lower limb during eight repetitions of a leg press [19]. For these authors, the more rigid (consistent) movement strategies displayed by the older adults were likely adopted because of an inability to control the multiple degrees of freedom present during the performance of a challenging coordination task [19]. When interpreting the results of this study, it is important to keep in mind that the variability of inter-joint coordination was explored during a closed-loop task, such as a leg press, while vertical jumping is an open-loop task. The addition of an equilibrium constraint during lower limb explosive movement might explain these different outcomes. Indeed, some research results on exercise indicate a positive relationship between the task (i.e., intensity, duration) and individual (i.e., physical, coordinative, and cognitive fitness) constraints to adjust motor coordination [48, 49]. It could be hypothesized that the observation of high variability in the squat jump task offers the system the flexibility to achieve stable balance in response to unstable external conditions [50]. Also, larger variability in older adults may be necessary to respond to a combination of age-associated changes in cognitive, sensorimotor, and physiological performance [51, 52]. Nevertheless, these results and the hypotheses posited require consideration to draw conclusions. A high level of CRP variability could be characterized as a “functional form of flexibility of the neuro-muscular system” observed in high-level athletes [50] as opposed to a “detrimental coordinative feature” [19, 47, 53]. These results may be addressed in terms of aging’s effect on explosive movement execution. Substantial evidence indicates that the neural controller takes advantage of motor redundancy (motor abundance) at the joint, muscle, and neural levels [54–57].

The main limitation of this study was the small sample size. The participant recruitment was limited to healthy men between 69 and 85 years of age for potential safety concerns with the jump protocol. Indeed, the choice of a healthy and active population does not provide a complete view of aging’s effects, only an optimal one. Longitudinal assessments of jump performance in a larger age range of participants are needed to complete this pilot study and to better understand the effect of aging on variability and coordination strategies.

## Conclusion

This study found that elderly compared to young participants demonstrated an altered inter-joint coordination during squat jumping despite a preserved proximodistal pattern. These results also displayed a higher variability in inter-joint coordination strategy in seniors. Due to the high explosive demand of the squat jump task, it could be hypothesized that this is a strategy used by the neuromuscular system to compensate for strength deficits or improve stability in seniors.

These findings have important implications for the interventions provided to older adults to preserve physical performance and functional abilities. The results suggest a benefit in the practice of explosive exercises in open-loop tasks to improve the central nervous system’s ability to cope with the control of complex tasks.

## Supporting information

S1 Dataset Vertical jump height dataset. Vertical jump height of young and elderly group.
(XLSX)

S2 Dataset MACRP and Variability of CRP dataset. Mean absolute continuous relative phase and continuous relative phase variability of the young and elderly groups.
(XLSX)
